# Valedictory Address to the Graduates of the Medical Department of Pennsylvania College—Session of 1816-47

**Published:** 1847-05

**Authors:** Washington L. Atlee

**Affiliations:** Prof. of Chemistry


### EDITORIAL DEPARTMENT.

---

*Valedictory address to the Graduates of the Medical Department of Pennsylvania College—Session of* 1846–47. By WASHINGTON L. ATLEE, M. D., Prof. of Chemistry.

Philadelphia has become the Salernum of Medical Literature and with her Medical Presses and Medical Colleges, she is now prepared to supply the whole continent with science and Doctors.—The bookmakers continue to pour out their volumes in endless series, and the professors continue to wheel up their fresh cohorts of doctors by battallions. If so many more are necessary to carry on this war with death, we are glad, at least, that the right men are employed in the recruiting service—men of high morals, and men of honor. Such a man we believe the author of the following sentiments to be.

"In your intercourse with your patients, observe candour and sincerity—truthfulness of character, is a jewel in every section of life. But in the Physician it is beyond price." * * * * "Be careful, also gentlemen, that the dignity of the profession does not suffer by retailing to the world the occurrences of the sick chamber." * * * * * * * * * * *

"Finally, gentlemen, in all your walks through life, *observe a proper degree of humility*. This feeling will never permit you to over-estimate your own powers; it will be continually leading you to self-improvement; it is the only true source of self-confidence; and may lead you to the greatest eminence. Observation proves that our most distinguished men are humble. Newton, in the midst of admiring friends, exclaimed, "to myself, I seem to have been as a child playing on the sea shore, while the immense ocean of truth lay unexplored before me." Socrates, in the depth and fervour of his philosophy, said he knew nothing. Luther, filled with zeal and piety, in the humility of his soul, confessed that he knew not the meaning of that word "Almighty." Peter the Great, descended from his high rank to work as a common labourer in a dock yard for the benefit of his empire. And Washington, glowing with the "amor patriæ," humbled himself on bended knee—even in the very pride and pomp of war—before the god of battles, to secure His blessings on our father's struggles for liberty. Simplicity of character is honorable, amiable and advantageous. The mind will thus always be open for knowledge from any source; it will always be ready to confess its ignorance; renounce its errors; and, if associated with energy, industry and perseverance, its elevation is beyond estimate. Genius, without these qualities, may glare like a meteor, and like a meteor will burst, suddenly shorn of all its glory; but even ordinary intellects, blessed with these redeeming virtues, though not so brilliant, are not so transient—like stars of the firmament, they throw their mellow and enduring light from one end of the world to the other forever."

We reccommend the discourse to all our readers.

